# Improving weather forecasting by assimilation of water vapor isotopes

**DOI:** 10.1038/s41598-021-97476-0

**Published:** 2021-09-14

**Authors:** Masataka Tada, Kei Yoshimura, Kinya Toride

**Affiliations:** 1 Japan Weather Association, 55F Sunshine City 60, Higashiikebukuro 3-1-1, Toshima-ku, Tokyo, 170-6055 Japan; 2 Institute of Industrial Science, The University of Tokyo, 5-1-5, Kashiwanoha, Kashiwa-shi, Chiba, 277-8574 Japan; 3 Department of Atmospheric Sciences, University of Washington, Seattle, WA 98195 USA

**Keywords:** Hydrology, Atmospheric dynamics

## Abstract

Stable water isotopes, which depend on meteorology and terrain, are important indicators of global water circulation. During the past 10 years, major advances have been made in general circulation models that include water isotopes, and the understanding of water isotopes has greatly progressed as a result of innovative, improved observation techniques. However, no previous studies have combined modeled and observed isotopes using data assimilation, nor have they investigated the impacts of real observations of isotopes. This is the first study to assimilate real satellite observations of isotopes using a general circulation model, then investigate the impacts on global dynamics and local phenomena. The results showed that assimilating isotope data improved not only the water isotope field but also meteorological variables such as air temperature and wind speed. Furthermore, the forecast skills of these variables were improved by a few percent, compared with a model that did not assimilate isotope observations.

## Introduction

Extreme weather is associated with natural disasters, and it impacts energy consumption and economics^1^. Improvements in weather forecasting can help mitigate the impact of climate change on global water resources, which is projected to increase^2–4^. In this study, the impacts of water vapor isotopes on weather forecasting were investigated using satellite observations. These isotopes are used as tracers to study the Earth’s water circulation in hydrology, meteorology, and climatology applications^5–7^.

Water vapor isotopes are sensitive to phase transition, and heavier molecules such as H^2^HO or H_2_^18^O preferentially condense/less preferentially evaporate, compared with ordinary H_2_O. The spatiotemporal variability of isotopes depends on these characteristics. This unique behavior is called the stable isotope effect, which was formulated empirically by Dansgaard^8^. Dansgaard found that the geographic distribution of isotopes in precipitation is related to latitude, air temperature, altitude, precipitation, and distance from the coast.

These unique characteristics have been noted, and observations have been collected for geoscientific research since 1961^9–11^ in the Global Network of Isotopes in Precipitation (GNIP) by the International Atomic Energy Agency (IAEA). However, the GNIP has only several hundred stations worldwide and observes isotopes in surface precipitation, which holds integrated information about various atmospheric processes. These data are both spatially and temporally much sparser than conventional non-isotopic data (e.g., wind, air temperature, and pressure). Recently, the performance of satellite-based spectrometers and the algorithms used to retrieve isotopes in atmospheric water vapor have both been improved^12–15^, and the improved versions are more directly sensitive to atmospheric processes. Previous studies indicated that δ^2^H observations provide an understanding of moisture processes due to isotopic fractionation^16–19^. Such studies include the estimation of condensation efficiency in convective activity^12,16^, the transport processes of dry air masses in the Sahel^13^, constraining vertical water vapor transport to reduce the moisture bias of models in the mid and upper troposphere^17,18^, and investigations of the hydrological cycle associated with large-scale convective reorganization^19^.

In parallel with enhancements in observation technology, numerical models that incorporate water isotopes have been developed and improved^20–30^. These models calculate the distribution of isotopes and provide information on the circulation of water isotopes in the absence of observations. Using an isotope-enabled numerical model, a past study investigated the impacts of assimilating water isotopes in idealized experiments^31^. Although the results indicated that atmospheric fields can be improved by assimilating water vapor isotopes, satellite-based observations such as those from the Tropospheric Emission Spectrometer (TES) and the Scanning Imaging Absorption Spectrometer for Atmospheric Cartography (SCIAMACHY) had less robust impacts than did mock in situ observations because of differences in the number of observations.

In this study, we investigated the utility of water vapor isotope data derived from the Infrared Atmospheric Sounding Interferometer (IASI)^14^, which has a much greater spatial and temporal observation coverage with improved accuracy, compared with TES and SCIAMACHY. IASI observes water vapor isotopes in the mid-troposphere globally twice per day. We evaluated the impact of assimilating IASI isotope data on various atmospheric fields and forecast accuracy.

## Methodology

### Assimilation scheme

We used a local ensemble transform Kalman filter (LETKF) for our data assimilation scheme. The Kalman filter (KF) estimates optimal values using a linear minimum variance estimation considering the covariance in both observation and model errors. The LETKF has a high calculation efficiency because of the localization technique^32^. The model error covariance is assumed to be represented by the covariance of a limited number of ensemble members. In our study, we used 30 ensemble members.

The analysis field, ${\text{x}}^{{\text{a}}}$, a matrix that serves as the initial conditions, is calculated by a linear combination of first guess and observation values as follows:

1 $${\text{x}}^{a} = {\text{x}}^{f} + {\text{K}}\left( {{\text{y}}^{ \circ } - {\text{H}}\left( {{\text{x}}^{f} } \right)} \right),$$

where ${\text{x}}^{{\text{a}}}$ consists of all prognostic variables of the model (zonal and meridional wind speed, air temperature, specific humidity, surface pressure, and hydrogen isotope ratio) at all model grids (192 × 94 × 28) for all 30 ensemble members; ${\text{x}}^{f}$ is the first guess (i.e., the forecasted field from the analysis of the previous cycle; 6 h ago in our study); K is the Kalman gain; *y*° is the IASI observation; and *H* is the observation operator that projects the Isotope-incorporated Global Spectral Model (IsoGSM) model field to the IASI observations field. Here, *H* is the spatial interpolation from the IsoGSM grids to the locations of IASI observations. The satellite averaging kernels were not applied to the model output in this study. The second term on the right side is the analysis increment, which indicates the degree of modification from the first guess.

### Observation data

We used IASI data for the data assimilation. Although our IASI data had 10 vertical layers, we assimilated only the fifth layer (at the height of approximately 4.5 km from the surface) because it contained the most reliable δ^2^H measurements^14^. Horizontal spatial coverage of IASI δ^2^H observations is from 60° S to 60° N. IASI makes approximately 1.3 million measurements per day, but only cloud-free measurements can be used to retrieve δ^2^H estimates. After removing obvious outliers and resampling to model resolution, nearly 230,000 points for April 2013 were used in the assimilation. The spatial distribution of δ^2^H observation points and monthly averaged data are shown in Fig. S1.

### Numerical model

We used IsoGSM as our numerical model with T62 horizontal resolution (approximately 200 km) and 28 vertical sigma levels^25^. Gaseous forms of heavier isotopologues (H^2^HO and H_2_^18^O) are incorporated into IsoGSM as prognostic variables, in addition to ordinary water vapor. All water isotopes are advected and transported by atmospheric circulation processes and subgrid-scale processes (convection and boundary layer turbulence). In addition, the fractionation during phase transitions, such as condensation or evaporation of precipitation, is also formulated in detail. The model’s physics packages include the longwave and shortwave radiation scheme of Chou and Suarez^33^, relaxed Arakawa–Schubert convective parameterization^34^, nonlocal vertical diffusion^35^, mountain drag^36^, shallow convection^37^, and the Noah land surface scheme^38^.

IsoGSM has been widely used in previous studies^39^. For example, IsoGSM was used to reproduce spatiotemporal variation in the isotopic field for the past 30 years^25^ by nudging with data from the National Centers for Environmental Prediction/Department of Energy (NCEP/DOE) reanalysis^40^. We used this dataset (referred to as isotopic reanalysis) to validate the assimilation experiments in this study because it has comparable accuracy to that of the NCEP/DOE reanalysis in terms of synoptic-scale fluctuation. The IsoGSM is one of few available isotopic reanalyses, and has been well validated with satellite measurements of water vapor isotopes^41^.

### Experimental setup

Our first experiment was conducted from 1 April to 6 May 2013 because of IASI data availability. The last 6 days (i.e., 1–6 May 2013) were considered the pure forecast period. Our second experiment was from 1–28 April 2013, and 23–28 April was used as the pure forecast period (Fig. S2). The calculation process is shown in Fig. S3. First, we generated 30 ensemble members with perturbed initial conditions using the lagged average forecast method^42^. Second, we made first-guess ensemble forecasts using IsoGSM. Third, we estimated the analysis fields based on the first guess and observations using the LETKF. Finally, we repeated the second and third processes. Note that IsoGSM and the LETKF are independent. IsoGSM simulated the mixing ratios of H^2^HO in the air (hereafter *q*_H2HO_) as a prognostic variable, and we converted these ratios to the isotope ratio of water vapor (hereafter δ^2^H) before the assimilation process as shown in Figure S3. This approach was used because mixing ratios have a lower bound of zero, which often violates the Gaussian assumption in LETKF. This conversion has also been employed in previous studies^39,43^. We decided to use 30 ensemble members, given the results of idealized experiments (data not shown).

Importantly, our non-assimilation experiment was a set of realizations of the atmosphere initialized from different dates in the natural run. Therefore, we considered the non-assimilation experiment to be independent of the truth; we considered the ensemble average of the non-assimilation experiment to be closer to a climatological condition.

In this study, we evaluated the accuracy of the analysis/forecast field based on changes in root mean square errors (RMSEs) between the assimilation and non-assimilation experiments. Specifically, we used the analysis/forecast skill defined by the difference between RMSE_DA/FC_ and RMSE_NA_, or the difference normalized by RMSE_NA_, defined as:

2 $${\text{Analysis}}/{\text{forecst}}\;{\text{skill}} = \frac{{{\text{RMSE}}_{{{\text{DA}}/{\text{FC}}}} - {\text{RMSE}}_{{{\text{NA}}}} }}{{{\text{RMSE}}_{{{\text{NA}}}} }} \times 100,$$

where RMSE_DA/FC_ is the RMSE of the analysis/forecast field against reanalysis data (isotopic reanalysis^25^ or ERA5^44^), and RMSE_NA_ is the RMSE of the non-assimilation experiment. Negative values indicate a higher accuracy than the non-assimilation experiment.

## Results and discussion

### The global impact of assimilating water vapor isotopes

**Figure 1:**
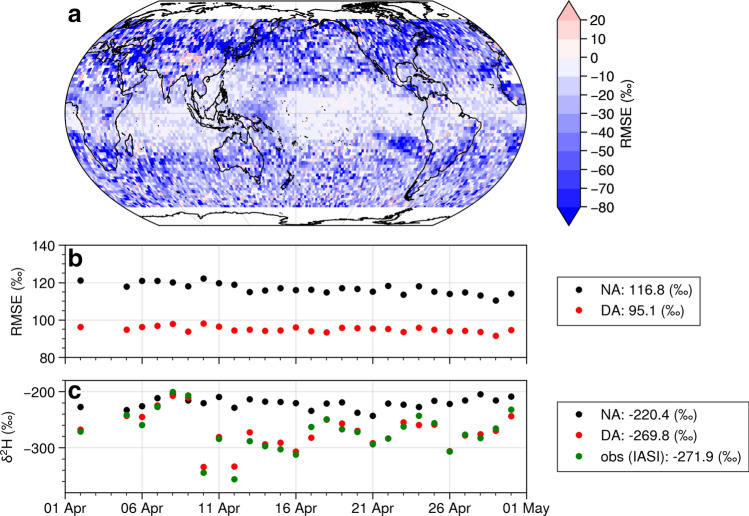
(**a**) Difference in root mean square error (RMSE) of 6-hourly δ^2^H in April for assimilation and non-assimilation experiments. Blue (red) indicates better (worse) performance when assimilating water isotopes. (**b**) 6-hourly time series of globally averaged RMSE of δ^2^H using IASI observation data from April 2013 at the assimilation altitude of approximately 4.5 km. (**c**) Time series of δ^2^H over Japan and the surrounding area where analysis skill was substantially improved for the middle troposphere. Black points indicate non-assimilation experiment, red points indicate assimilation experiment, and green points indicate IASI observations. Black and red points are collocated averages of IASI observations. Figure created using Python 3.7.6 (https://www.python.org/).

We conducted experiments with and without assimilating IASI data for 1–30 April 2013. Figure 1 shows the impacts of assimilating water vapor isotopes. The analysis skill improved, especially in mid-latitudes (Fig. 1a) due to the high number of observations. Similarly, a greater improvement was observed in the Northern Hemisphere than in the Southern Hemisphere. This was because of the difference in available observations and the accuracy of observed hydrogen isotopes. A previous study found larger observational errors at middle and high latitudes in the winter hemisphere^45^.

However, improvements were limited at low latitudes, although many observations were assimilated (Fig. S1a). This was probably because of the poor consistency between modeled and observed isotopic behavior at low latitudes due to the poor representation of the latitude effect associated with the convective process, as demonstrated by a previous study comparing modeled isotopes with TES and SCIAMACHY data^41^.

Figure 1b shows 6-hourly time series of globally averaged RMSE of δ^2^H using IASI observation from April 2013 at the assimilation altitude of approximately 4.5 km. During the study period, the isotope assimilation improved RMSE by at least 10% globally. We further investigated how assimilating water vapor affected other variables on a global scale. Figure S4 shows the vertical profile of the normalized change in RMSEs between the two experiments, using the isotopic reanalysis as a reference. Although assimilating water isotopes improved most variables globally throughout most of the troposphere, the profile of improved skills differed among variables. Furthermore, we compared the results with an ERA5 reanalysis (i.e., the ECMWF atmospheric reanalysis in Fig. S5). The results indicated almost the same behavior as in the isotopic reanalysis, confirming the positive effects of isotope assimilation on various atmospheric fields. Notably, the ERA5 data were spatially interpolated to match the resolution of IsoGSM.

Specific humidity and air temperature were improved at the assimilation altitude (4.5 km), because these variables are directly related to water vapor isotopes through isotopic fractionation. The analysis skills for geopotential height, zonal wind speed, and meridional wind speed were not necessarily improved at 4.5 km altitude, but they were improved overall for the rest of the troposphere. This was because variables not directly related to isotopes are indirectly improved by other variables directly related to isotopes, such as air temperature. Thus, water isotopes hold integral information about the complex water cycle and the dynamics of moisture transport.

### Local impacts of assimilating water vapor isotopes

Figure 1c shows the time series of regionally averaged δ^2^H for Japan and the surrounding area, a region where analysis skill was particularly improved for the mid-troposphere (Fig. 1a). In spring, the weather in Japan tends to change substantially due to the alternate passage of low-pressure systems and traveling high-pressure systems^46^. Assimilating water vapor isotopes helped represent these changes due to the abundant observations of water vapor isotopes in and upwind of Japan (Fig. S1a). The analysis skill was not necessarily improved at points with a large number of observations (Fig. S6a) because of the compound impacts of data assimilation and local meteorology (advection and convection).

**Figure 2:**
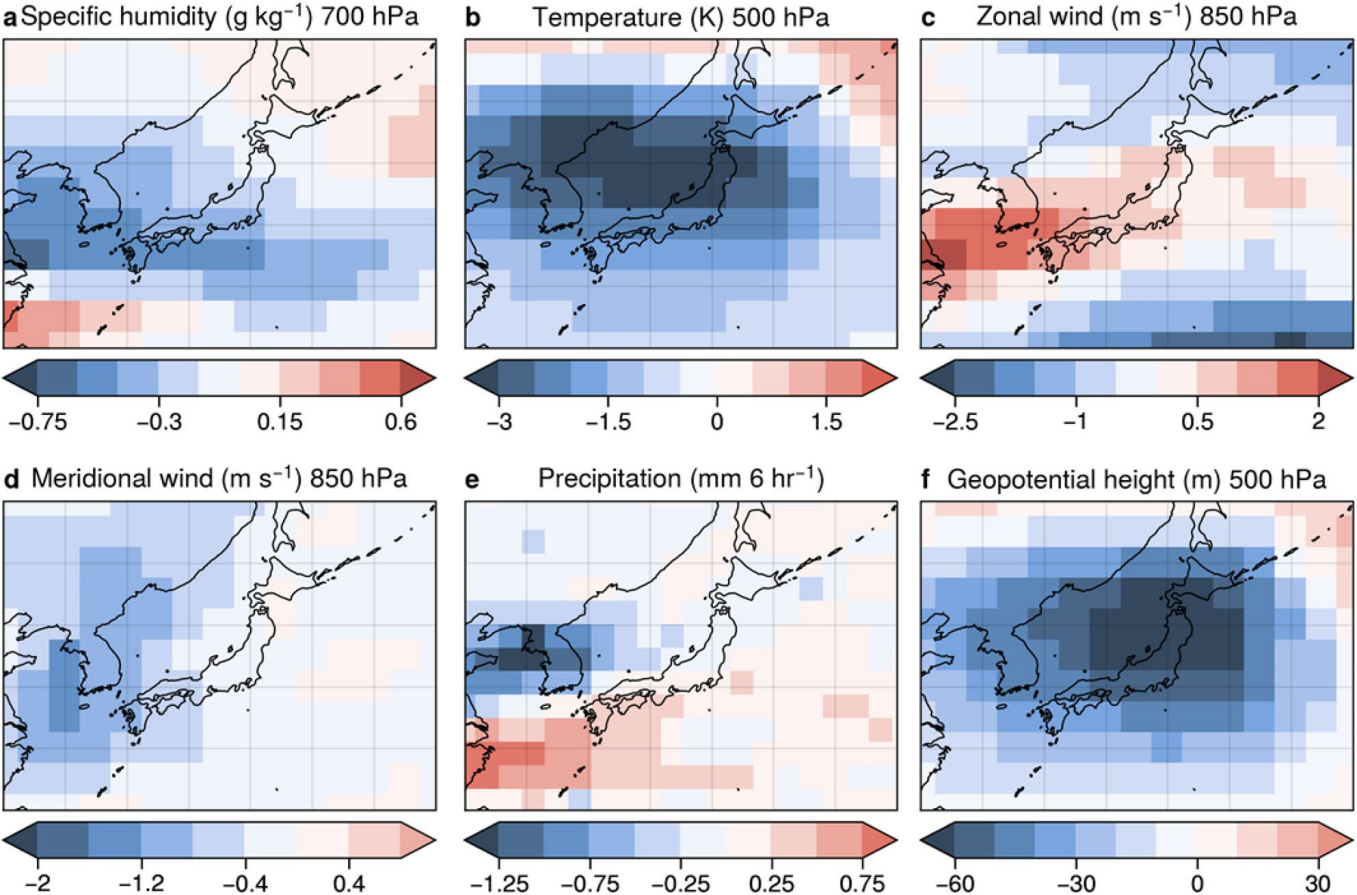
Difference in RMSEs between assimilation and non-assimilation experiments during April 2013 over Japan for (**a**) specific humidity, (**b**) temperature, (**c**) zonal wind, (**d**) meridional wind, (**e**) precipitation rate, and (**f**) geopotential height. The RMSE is derived from a 6-hourly time series with the isotopic reanalysis as a reference. Blue is better, red is worse (for the assimilation experiment). If the number is negative, the assimilation experiment was better than the non-assimilation experiment. Figure created using Python 3.7.6 (https://www.python.org/).

Figure 2 shows the difference in RMSE derived from 6-hourly time series between assimilation and non-assimilation experiments using the isotopic reanalysis over Japan. Assimilating water isotopes had positive effects on specific humidity, air temperature, and winds (Fig. 2a–d,f) because water isotopes are sensitive to these variables. In contrast, the precipitation rate showed a deterioration for the domain average.

Specific humidity was improved over Japan and on the west side of Japan, whereas it worsened on the south and north sides (Fig. 2a). The zonal wind worsened over Japan and on the west side of the country, contrary to the specific humidity; it improved on the south and north sides of Japan. (Fig. 2c). The zonal wind was improved over a large part of this area, but it deteriorated slightly over the eastern seas of Japan (Fig. 2d). Because water vapor isotopes are inherently sensitive to precipitation processes, the precipitation-rate was improved to the north of the domain, where the numbers of observations were large (Fig. 2e). Assimilating water vapor isotopes generally had a positive impact in rainy regions such as from western China to the north of Japan, the sea to the north of Australia, from the Bay of Bengal to the south of the Philippine Sea, and the sea to the east of the North American continent (not shown). In contrast, geopotential height at 500 hPa may not have a direct relationship with isotopes; however, because of the close relationship with air temperature, improving the isotopic field resulted in an improvement in geopotential height. We also made the same comparison using ERA5 (Fig. S7). Overall, the results were similar to the findings of the isotopic reanalysis. This indicated the robustness of our results, regardless of the choice of reanalysis datasets.

### Focus on a low-pressure event over Japan and the surrounding area

Here, we investigated a case study of a low-pressure event over Japan on 19 April 2013 associated with precipitation. Considering the resolution of the model and the characteristics of the isotope effect, we chose a large-scale event to evaluate the impacts of assimilating water isotopes, rather than a small and short phenomenon (e.g., a convective disturbance). As explained in Sect. 2.4, the non-assimilation experiment was a set of free ensemble forecasts starting from different initial conditions, such that the performance was closer to a climatological condition and it could not capture any particular events by definition.

**Figure 3:**
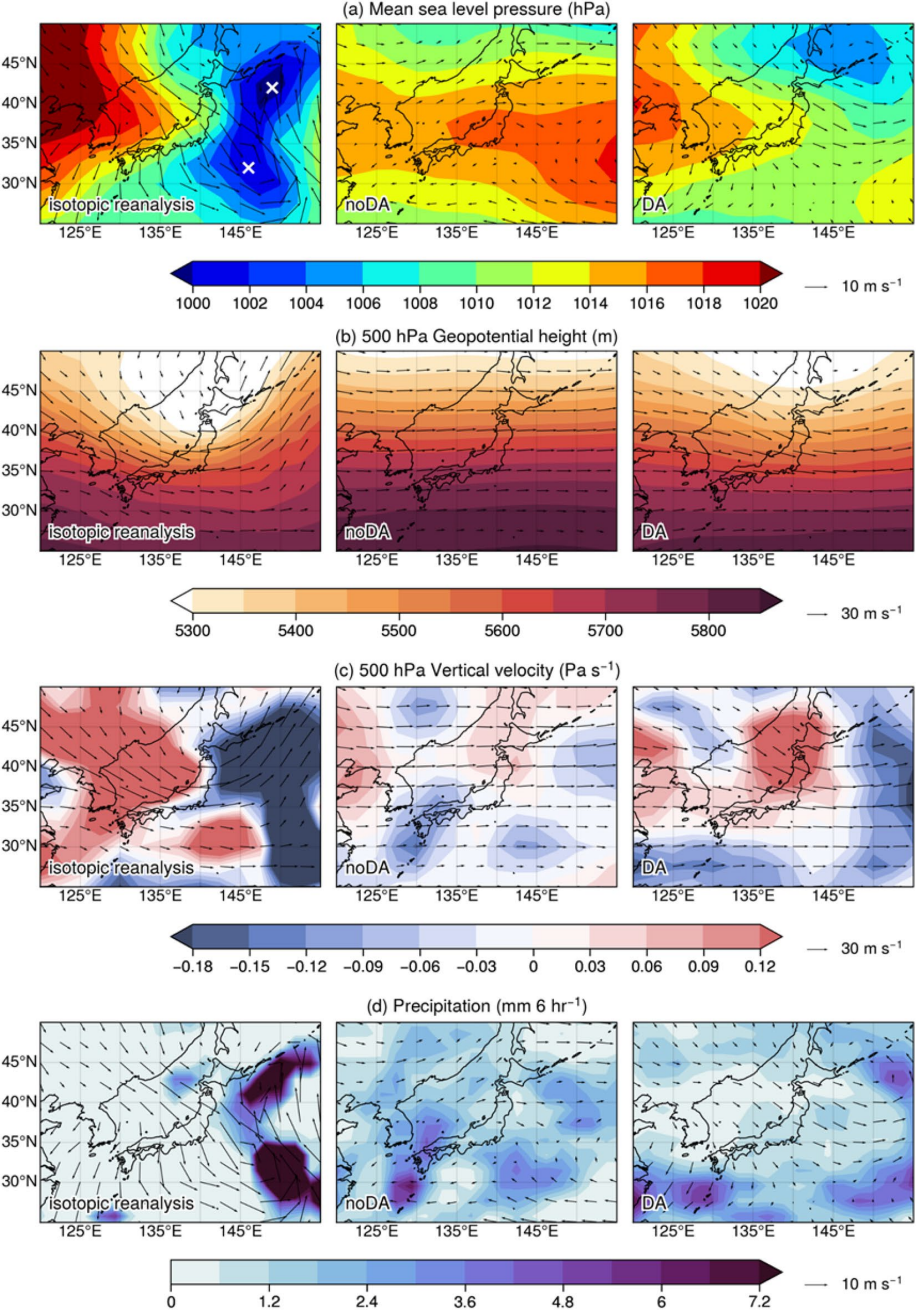
Snapshot of a low-pressure system case study for Japan and the surrounding area on 19 April 2013 at 00:00 UTC for (**a**) 500 hPa pressure reduced to mean sea level (MSL; the two white stars indicate the locations of the two low-pressure centers), (**b**) 500 hPa geopotential height, (**c**) vertical velocity at a height of 500 hPa, and (**d**) precipitation rate. The orientation and length of the arrows indicate wind direction and speed (m s^-1^), respectively. The number below the arrow in the legend for each panel indicates the wind speed for that arrow length. Figure created using Python 3.7.6 (https://www.python.org/).

The left column of Fig. 3 shows the isotopic reanalysis fields, in which two low-pressure centers were observed over eastern Japan (locations marked with white stars in Fig. 3a). We defined the centers of these low-pressure systems using mean sea-level pressure and wind circulation: northern low near 42°N and 150°E, and southern low near 32°N and 147°E. Corresponding to the low-pressure centers, a thermal trough was located over Japan (Fig. 3b), and downward (upward motion) existed to the west (east) of the thermal trough (Fig. 3c). As expected, rainfall occurred near the northern and southern lows (Fig. 3d), and winds at 500 hPa height blew along isobars while surface winds indicated cyclonic circulation near the low-pressure centers.

The experiment with no isotope assimilation did not model the two low-pressure centers; however, the experiment that assimilated the water vapor isotopes was able to simulate the northern low pressure (Fig. 3a). The southern low pressure was not modeled as clearly, but a lower pressure area could be observed in the south of Japan, which indicates that assimilating water vapor isotopes helped to produce the overall pressure structure for the region. The difference in the ability to model the northern and southern lows indicates weak assimilation constraints by the isotopes alone. However, the ability to model the overall pressure pattern simply by incorporating water vapor isotopes in the assimilation suggests the high potential of providing additional constraints when combined with conventional observations. This possibility was demonstrated in recent research, with idealization experiments assimilating traditional meteorological measurements^42^. Furthermore, the pressure field in the assimilation experiment was more similar to the isotopic reanalysis in terms of high pressure to the west. As explained in Sect. 3.2, the improvements in geopotential height were likely associated with improvements in air temperature because of the strong relationship between isotopes and temperature.

The thermal trough was completely absent in the non-assimilation experiment, but in the assimilation experiment, a weak trough was produced at a similar location as in the isotopic reanalysis (Fig. 3b). Vertical velocity was completely different in the non-assimilation experiment and the isotopic reanalysis. The assimilation experiment, however, was more accurate in terms of descending air occurring ahead of the low pressure and ascending air occurring near the center of the low pressure (Fig. 3c).

In the non-assimilation experiment, precipitation occurred in regions not corresponding to the low-pressure centers; in the assimilation experiment, precipitation occurred over the areas corresponding to the lows (Fig. 3d). The assimilation grid in this region is shown in Fig. S6b. Although only the results over Japan are mentioned here, precipitation was improved over Japan and across many regions on a global scale, as discussed in Sect. 3.2. The wind field generally resembles the field of geopotential height because the wind blows along isobars at a synoptic scale. At the 500-hPa level, the westerly wind blew zonally in the non-assimilation experiment, but it blew along the analyzed thermal trough in the assimilation experiment. There was no cyclonic circulation in the non-assimilation experiment near the surface, but circulation corresponding to the northern low was evident in the assimilation experiment (Figs. 3a and d).

Model bias and model characteristics could not be completely eliminated, but we found that isotope information overall improved synoptic-scale pressure patterns and other fields such as temperature, wind, and precipitation.

### Sensitivity to assimilation period

We conducted two 6-day forecast experiments to evaluate whether water vapor isotopes can improve atmospheric forecasts. Assimilating water vapor isotopes improved many variables throughout the troposphere during the analysis period. We conducted these two experiments to investigate how much forecast skill changes for different assimilation periods. We compared the forecast after 30 days of assimilation with the forecast after 22 days of assimilation.

**Figure 4:**
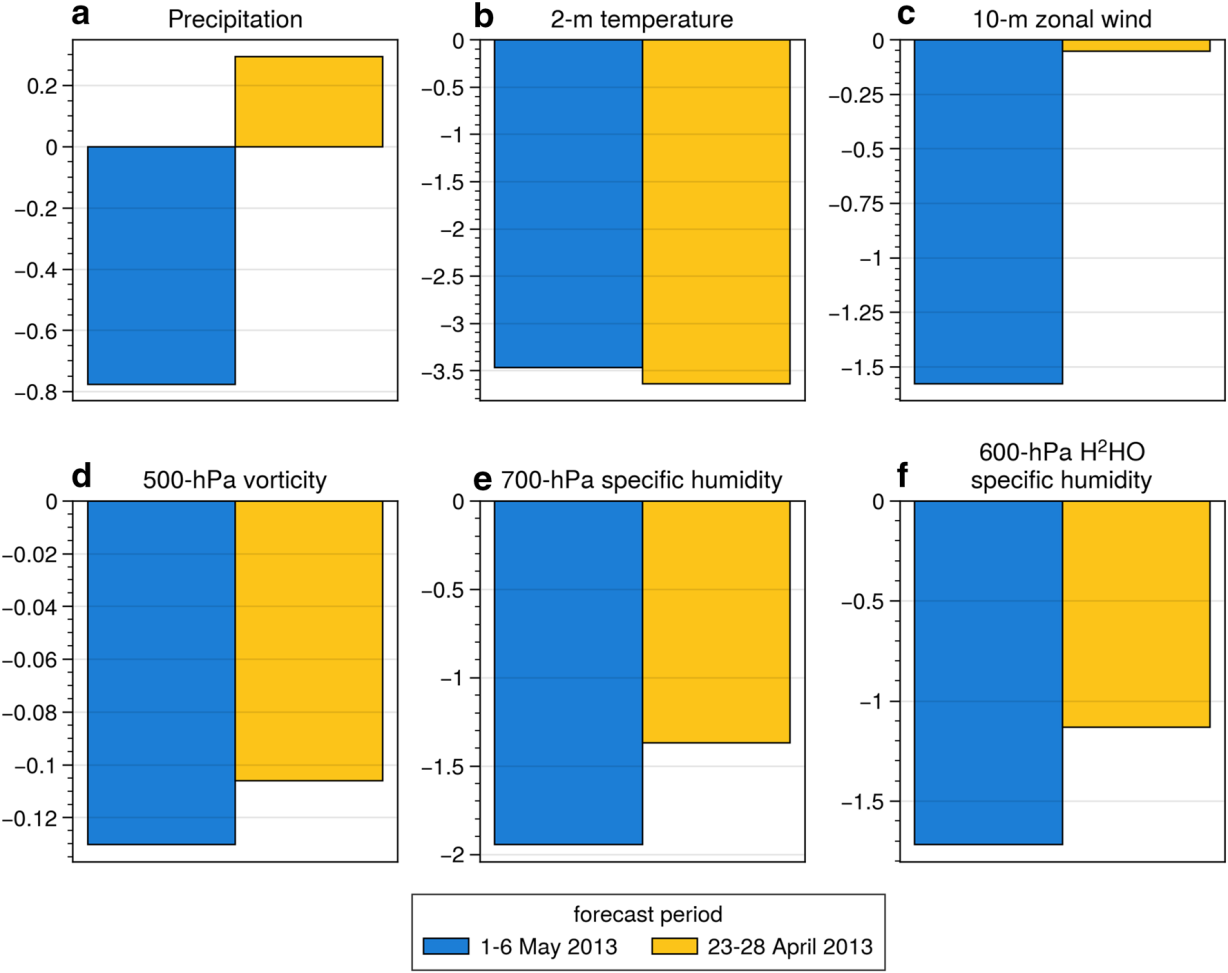
Comparison of the two assimilation experiments on improvement in forecast skill over the non-assimilation experiment for (**a**) precipitation rate, (**b**) 2-m temperature, (**c**) 10-m zonal wind, (**d**) 500-hPa vorticity, (**e**) 700-hPa specific humidity, and (**f**) 600-hPa H^2^HO specific humidity. Forecast skill is defined as the normalized difference in global RMSEs between assimilation and non-assimilation experiments during the forecast period. Blue bar indicates the assimilation experiment with the forecast period 1–6 May 2013. Yellow bar indicates the assimilation experiment with the forecast period 23–28 April 2013. The unit for the y axes is %. Negative values indicate that the assimilation experiment was better than the non-assimilation experiment. Figure created using Python 3.7.6 (https://www.python.org/).

Figure 4 shows a comparison of the two assimilation experiments in terms of forecast skill against the non-assimilation experiment. The blue bar indicates the assimilation experiment with a forecast period of 1–6 May 2013. The yellow bar indicates the assimilation experiment with a forecast period of 23–28 April 2013. Although the assimilation periods and the prediction times differed, the assimilation forecast skill was better than the non-assimilation forecast skill for many variables. In particular, air temperature forecast skill was improved by approximately 3.5% in both forecast experiments (Fig. 4b). Furthermore, more improvements were obtained with assimilation for 30 days than with assimilation for 22 days, in particular for precipitation and zonal wind (Fig. 4a, c–f). As previous studies indicated, a longer assimilation period was closer to the truth during the spin-up period^45^. Our results demonstrated that the accumulation of isotope observations helps improve weather forecasting.

## Summary and conclusions

We conducted experiments that directly assimilated real satellite observations of water vapor isotopes. Although these assimilated data were limited to 1 month, we demonstrated that assimilating water vapor isotopes is useful for modeling low-pressure centers on a local scale and for improving forecast skill out to 6 days on a global scale. Furthermore, a comparison with several reanalysis datasets showed the same tendency, indicating that the improvements obtained by assimilating water vapor isotopes were robust regardless of the choice of reanalysis datasets.

Importantly, there was a systematic bias between the model and observations. In general, the simulated latitudinal isotope ratio gradients were smaller than the observed latitudinal isotope ratio gradients^17,41^. A study comparing several isotope-enabled models, including IsoGSM, with in situ observations suggested that not all models accurately simulate the spatial structure of marine boundary layer isotopic composition^47^. Furthermore, to ensure accurate forecasting, the atmospheric state-dependent vertical information in the IASI data should be considered^48^. It is also important to study the impacts of assimilating water isotopes in addition to conventional observations. A recent study showed that the inclusion of water vapor isotopes could greatly contribute to weather forecasting using idealized experiments with synthetic water isotopes (based on IASI) and conventional observations (based on the NCEP operational system PREPBUFR)^43^. Real satellite observations should be implemented in this framework.

In this study, we only used IASI data, which are sensitive in the stratosphere and free troposphere. With the increasing number of wide-range and high-frequency observations by spectrometers and other instruments^49,50^, we believe that the use of water isotopes in addition to the traditional meteorological elements can improve the analysis and prediction accuracy of meteorological fields.

## Supplementary Information

Supplementary Information.

## Data Availability

The authors declare that all data generated or analyzed in the current study are available from the corresponding author on reasonable request.
