# Supplementary material for: Improving weather forecasting by assimilation of water vapor isotopes
Source: Sci Rep. 2021 Sep 14;11:18067. doi: 10.1038/s41598-021-97476-0 (PMC8440787; doi:10.1038/s41598-021-97476-0)
Supplement: Supplementary file 1 — Supplementary Information. [file 41598_2021_97476_MOESM1_ESM.docx]

**Supplementary Figures**

**
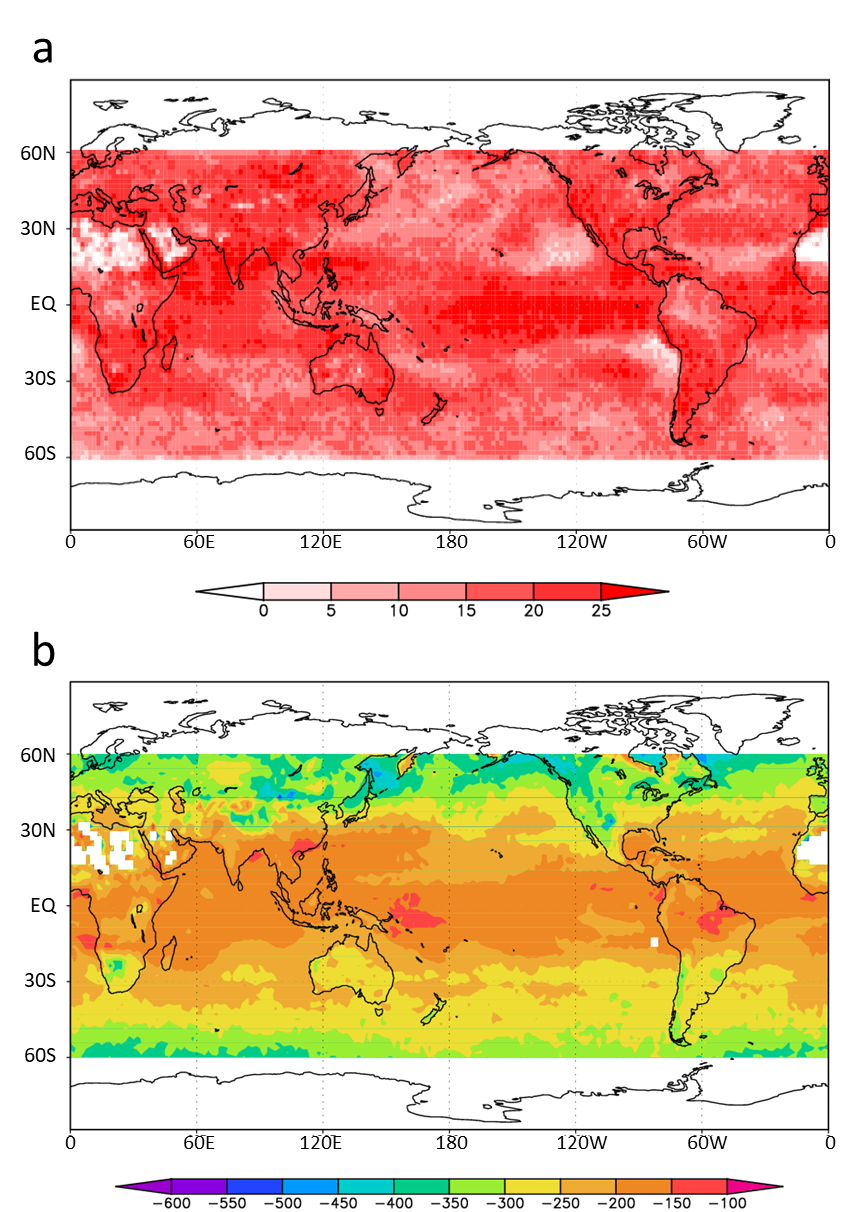
**

**Supplementary Figure S1** Information on assimilated observations. (a) Total number of assimilation points for April 2013. (b) Global distribution of 1-month averaged δ^2^H observations by IASI in April 2013. Figure created using GrADS 2.0.a5 (<http://cola.gmu.edu/grads/>).


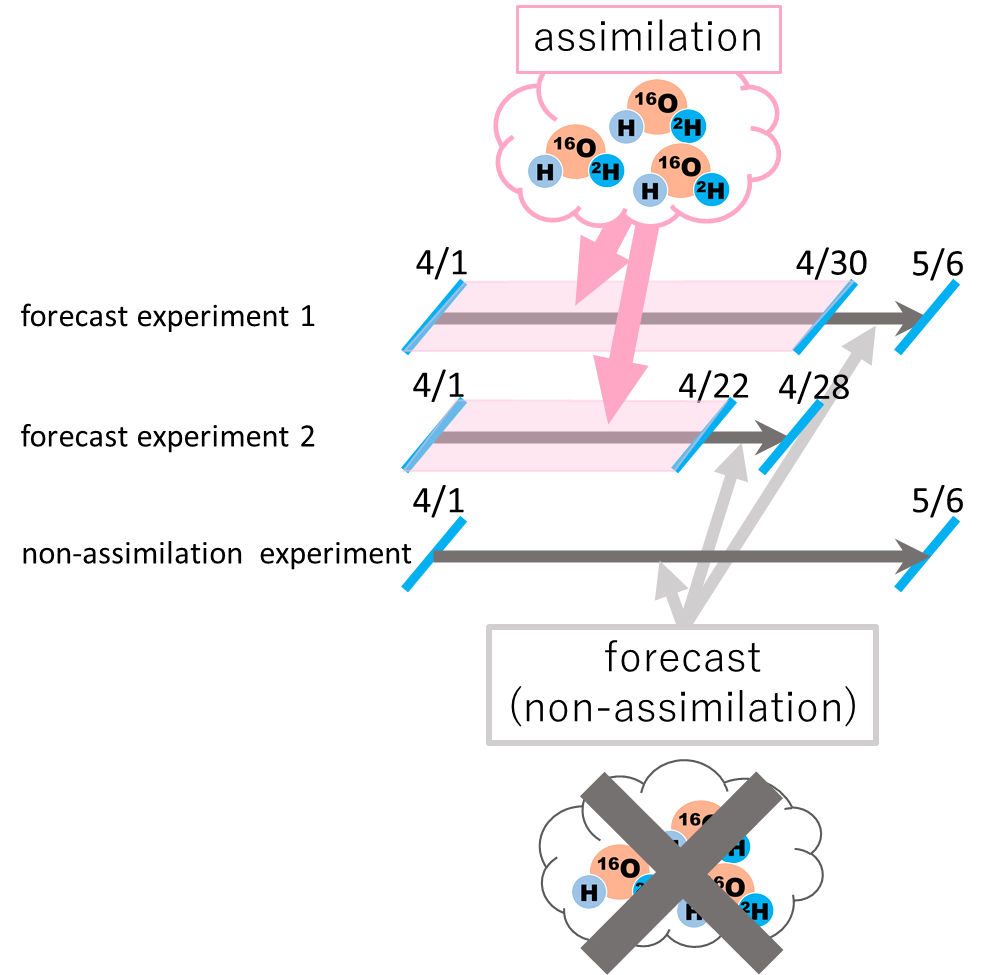


**Supplementary Figure S2** Experimental setup for the forecast experiments. Period with pink color indicates assimilation period and absence of pink color indicates forecast period. The first forecast experiment was conducted from 1 April to 6 May 2013, and the second forecast experiment was conducted from 1 April to 28 April 2013. The last six days were considered the pure forecast period. Non-assimilation experiment was conducted from 1 April to 6 May 2013. Figure created using PowerPoint for Windows.


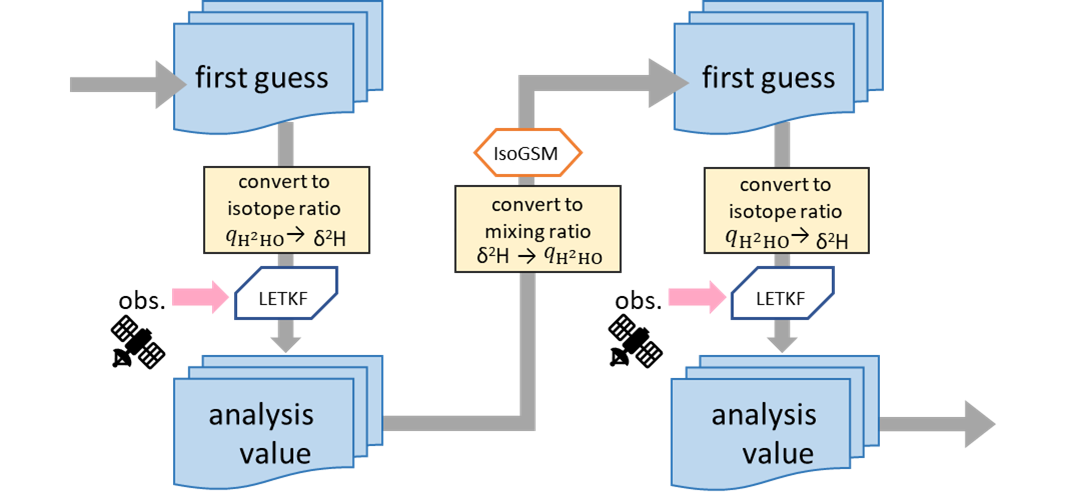


**Supplementary Figure S3** Flowchart of the assimilation process: (1) produce analysis values (initial values) by assimilating isotopes into first guess; (2) calculate next time step first guess value by inputting analysis into IsoGSM. Figure created using PowerPoint for Windows.


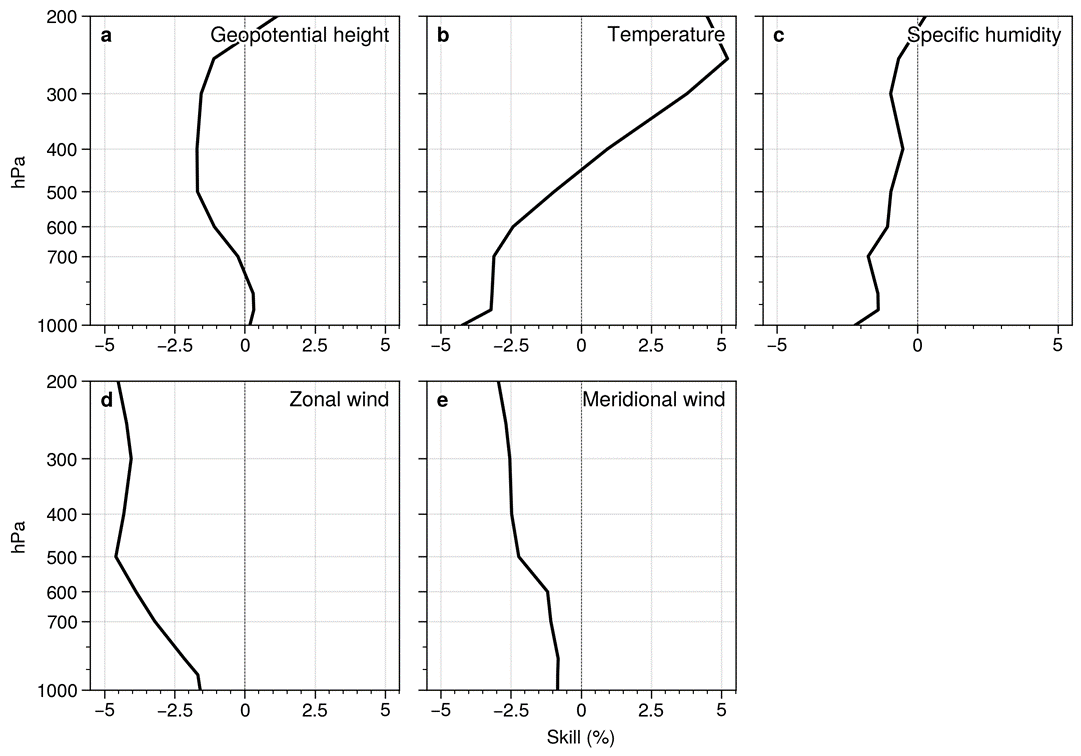


**Supplementary Figure S4** Vertical profiles of changes in normalized global RMSE values between the two experiments averaged over April 2013: (a), (b), (c), (d), and (e) indicate geopotential height, temperature, specific humidity, zonal wind, and meridional wind, respectively. Altitude (hPa) is on the y axis, and differences in normalized RMSE values between the two experiments for each variable are on the x axis (%). Negative values denote an improvement for the assimilation experiment. Red line denotes where the change in RMSE is zero. Figure created using Python 3.7.6 (<https://www.python.org/>).


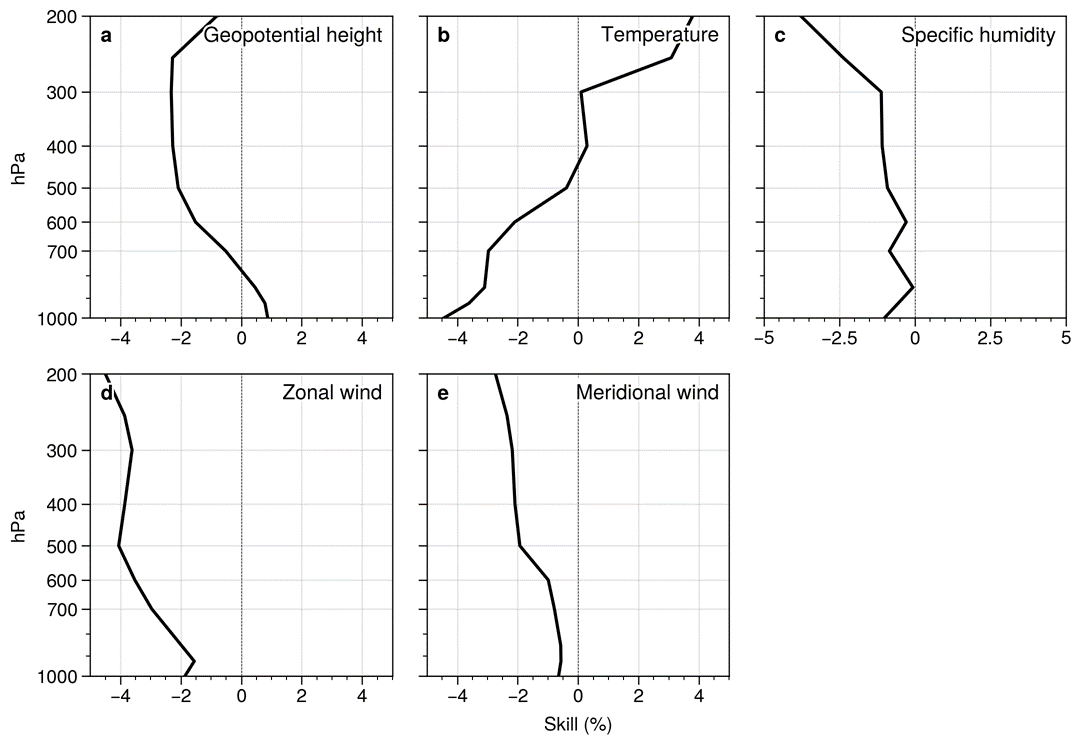


**Supplementary Figure S5** Similar to Supplementary Figure S4, but compared with reanalysis data in ERA5. Figure created using Python 3.7.6 (<https://www.python.org/>).


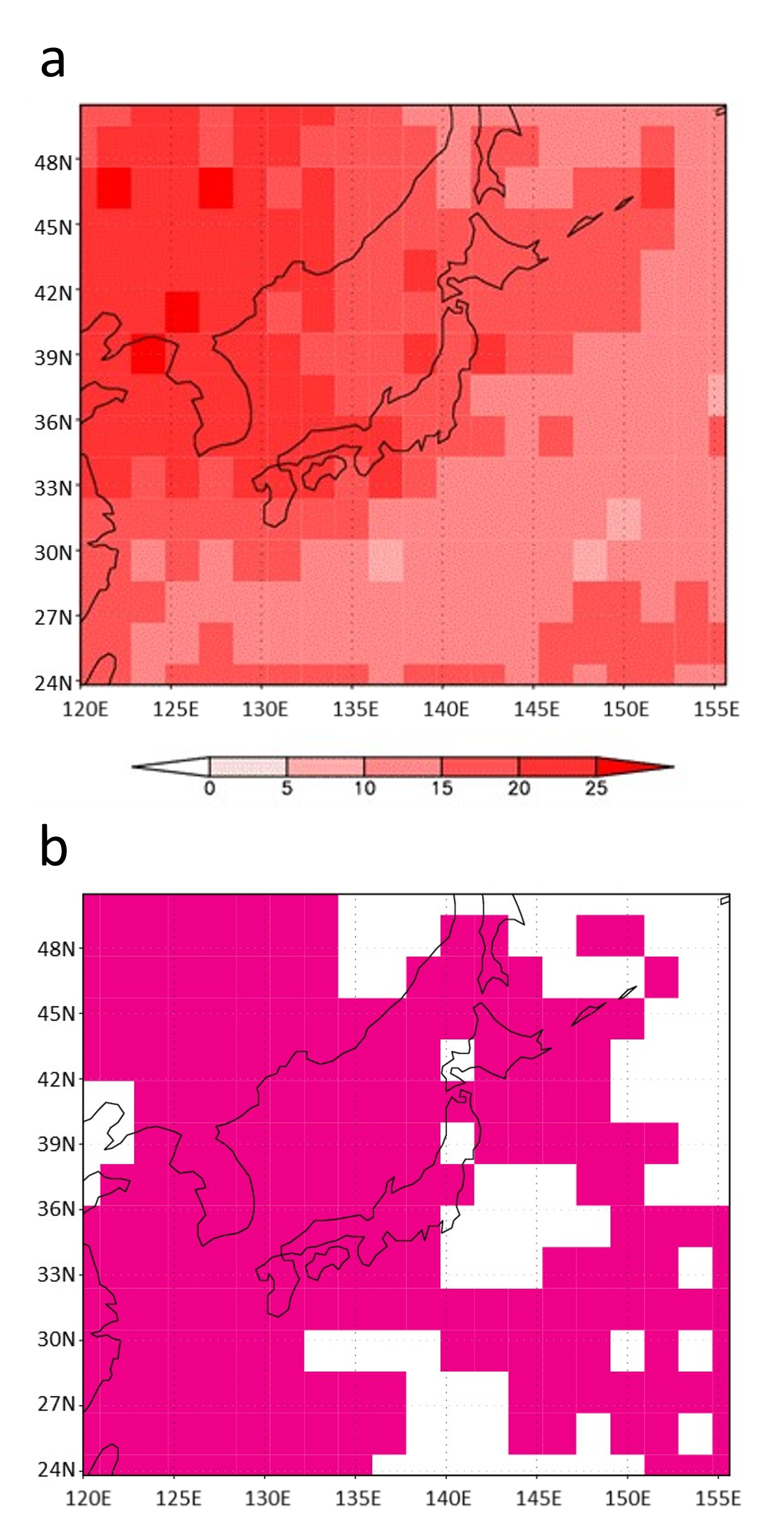


**Supplementary Figure S6** Assimilated observation points for Japan and the surrounding area: (a) for the whole of April 2013 and (b) for 19 April 2013 at 00:00 UTC. Shaded area in the grid indicates assimilated isotope information on 19 April 2013. Figure created using GrADS 2.0.a5 (<http://cola.gmu.edu/grads/>).


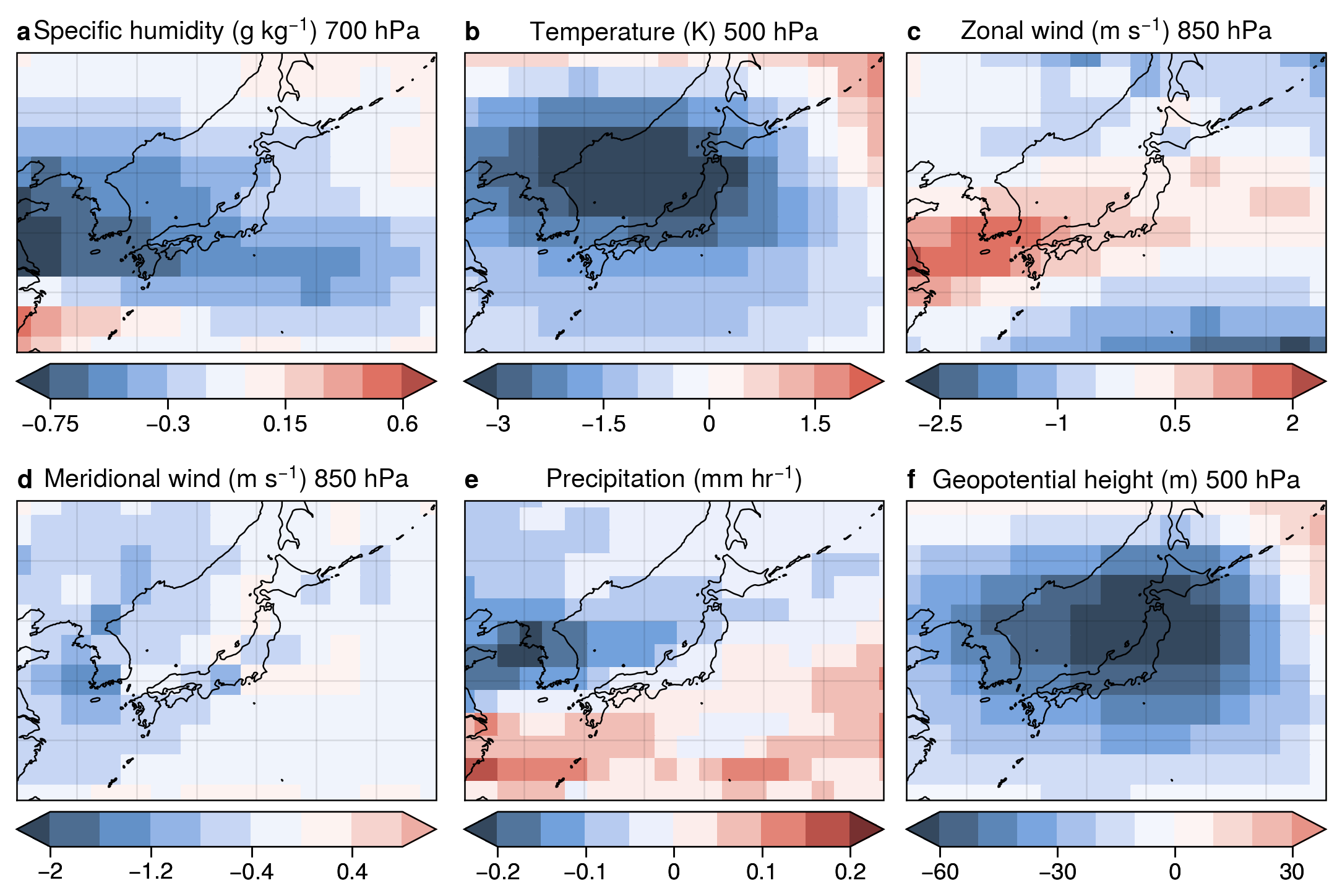


**Supplementary Figure S7** Similar to Figure 2, but compared with reanalysis data in ERA5. Figure created using Python 3.7.6 (<https://www.python.org/>)
